# The Huntington's Disease health-related Quality of Life questionnaire (HDQoL): a disease-specific measure of health-related quality of life

**DOI:** 10.1111/j.1399-0004.2011.01823.x

**Published:** 2012-02

**Authors:** MB Hocaoglu, EA Gaffan, AK Ho

**Affiliations:** Department of Psychology, School of Psychology and Clinical Language Sciences, University of ReadingReading, UK

**Keywords:** Huntington's disease, patient-reported outcome, pre-symptomatic/preclinical Huntington's disease, quality of life, questionnaire, HDQoL

## Abstract

Hocaoglu MB, Gaffan EA, Ho AK. The Huntington's disease health-related quality of life questionnaire: a disease-specific measure of health-related quality of life.

Huntington's disease (HD) is a genetic neurodegenerative disorder characterized by motor, cognitive and psychiatric disturbances, and yet there is no disease-specific patient-reported health-related quality of life outcome measure for patients. Our aim was to develop and validate such an instrument, i.e. the Huntington's Disease health-related Quality of Life questionnaire (HDQoL), to capture the true impact of living with this disease. Semi-structured interviews were conducted with the full spectrum of people living with HD, to form a pool of items, which were then examined in a larger sample prior to data-driven item reduction. We provide the statistical basis for the extraction of three different sets of scales from the HDQoL, and present validation and psychometric data on these scales using a sample of 152 participants living with HD. These new patient-derived scales provide promising patient-reported outcome measures for HD.

Section Editor: Aad Tibben, email: a.tibben@lumc.nl

The concept of health-related quality of life or the impact of health problems on personal well-being and life satisfaction (1) is particularly pertinent in neurodegenerative disorders where there is no known treatment to effectively modify the relentless progression of disease. Huntington's disease (HD) is a fully penetrant neurodegenerative disorder characterized by motor, cognitive and psychiatric disturbances that usually occur in mid-life (2). This multifaceted disorder begins insidiously and has a protracted course of up to approximately two decades. The all-encompassing multidimensionality of the quality of life concept makes it particularly relevant in chronic and degenerative disease (3, 4) such as HD. Therefore health-related quality of life is an important patient-reported outcome measure that will provide information on patients' personal everyday experiences. Due to the genetic basis and complex constellation of signs and symptoms of HD, the subsequent multidimensional impact on patients is great, and so it is important that a disease-specific measure is developed in order to fully capture the impact of living with HD. Data from our studies (5, 6) and others (7, 8) provide indications that generic scales are unlikely to convey the true impact of living with all aspects of HD, particularly the many non-physical aspects of this neurodegenerative disease.

The measurement of patient-reported outcomes is increasingly important in clinical trials because it provides the patients' point-of-view which reflects the overall net impact of any change or intervention; it takes into account adverse-effect profiles that differentially affect the various facets of disease, function and quality of life as experienced by patients from their unique perspective.

In this study, we report on the development and validation of the first patient-derived disease-specific health-related quality of life scale for HD that aims to adequately capture and measure the reported impact on everyday life in this population. This new disease-specific HD instrument, the Huntington's Disease health-related Quality of Life questionnaire (HDQoL), will provide validated patient-reported outcome measures suitable for capturing health-related quality of life in a way that is pertinent to this population.

## Methods and results

### Development of the HDQoL

#### Item generation from qualitative interviews

Semi-structured interviews were conducted in England with 31 people living with HD, from pre-symptomatic individuals to late-stage HD. Pre-symptomatic individuals were included since psychosocial and quality of life issues are relevant to this subgroup as well (9). Interview questions were developed after a review of literature, and included open-ended and specific probe questions which addressed the health-related quality of life definition adopted (emotional well-being, spirituality, sexuality, social functioning, family life, occupational functioning, communication, eating, functional ability, physical status, treatment satisfaction, self-esteem, body image, future orientation, global ratings of health and life satisfaction (10)). These face-to-face interviews were audio-recorded, transcribed and analyzed to identify points generated by participants, which then formed the basis for an initial pool of 81 items. Ethical approval for the project was obtained from the University of Reading Research Ethics Committee.

#### Item selection and questionnaire refinement

Feedback on these initial items was sought from a wider pool of people living with HD to aid data-driven item selection. Where necessary, these items were translated by two appropriately bilingual members of the European Huntington's Disease Network Quality of Life Working Group and/or their associates. The disease staging of patients was inferred via self-report responses to questions concerning functional ability (11). Feedback on these initial items was obtained from 281 people living with different levels of HD severity, including at-risk individuals with HD family history but have not undergone genetic testing, and pre-symptomatic individuals who were gene-positive carriers with no clinical motor signs of HD. Each HD severity subgroup had over 27 participants. Participants were from 12 different countries, i.e. England (34.3%), Canada (20%), Spain (9.8%), Scotland (9.5%), Belgium (8.3%), Norway (5.7%), Germany (4.4%), Switzerland (2.2%), Portugal (1.9%), Italy (1.6%), France (1.3%) and Ireland (0.6%).

Participants indicated how frequently they experienced difficulty with each item as a result of HD, and also how important each item was for their quality of life. Items with low importance scores, high omission rates or ambiguous content were eliminated. Item frequency scores of the remaining items were then examined using Rasch analysis; items located at a similar point on the logit scale were weighed against each other on semantic content, coverage and uniqueness. Analyses of variance procedures were also used to examine items in terms of disease severity stages.

### Validation of the HDQoL

The streamlined 40-item HDQoL questionnaire was edited and reworded to enhance clarity. The HDQoL was then pre-tested on a group of 20 participants from pre-symptomatic to late-stage disease and reviewed in the light of their feedback. The mean questionnaire completion time of the questionnaire, across all disease stages, was 22 min; all participants indicated that others in their position would be positively predisposed to completing this questionnaire, with 65% indicating it would be acceptable, and 35% highly acceptable.

Following this pre-testing, the revised HDQoL (available from the corresponding author), two generic health questionnaires, i.e. EuroQoL (EQ-5D) and SF-12v2, and a demographic information sheet with self-report on questions concerning functional ability (11) were posted to the Huntington's Disease Association United Kingdom members. Since membership was not fully updated and also did not distinguish between personal and professional members, a meaningful response rate could not be determined. Nevertheless, of the 261 questionnaires returned, 108 (41.38%) people decided not to participate in the study due to miscellaneous personal reasons, with fewer than 5% citing questionnaire difficulty. There were 152 completed questionnaire sets (Table 1) and 102 participants indicated that they would like to be contacted for the retest study 4–6 weeks later, where the same questionnaires were resent to participants, to establish test–retest reliability amongst participants with no change in health-related quality of life. Participants were instructed to complete the questionnaire from their own perspective, even if they might require physical assistance in filling the forms.

**Table 1:** Key demographic details of the validation sample

	No. of participants (*N* = 152)
*Self-reported HD stage*	
At risk	25
Pre-symptomatic gene positive	29
Stage 1	13
Stage 2	13
Stage 3	15
Stage 4	39
Stage 5	16
Information missing	2
*Sex*	
Male	52
Female	100
*Age group*	
Lowest to 44	35
44–64	85
65 to Highest	32
*Marital status*	
Married or living with a partner	104
Divorced or separated	23
Single	19
Widowed	5
Information missing	1
*Years of equivalent full-time education*	
0–6 years	4
7–12 years	54
13 to highest	63
Information missing	31

HD, Huntington's disease.

Since one of the aims of this study was to provide suitable health-related quality of life measures for this population, we recognize that the type of measure suitable varies depending on the purpose of investigation. We therefore provide the statistical basis for the extraction of three different sets of scales and their corresponding psychometric properties, based on (i) Primary Scales from a robust core triad of factors underlying health-related quality of life, (ii) Specific Scales from a more detailed profile of six clinically meaningful dimensions, and finally (iii) a Summary Scale.

#### Three Primary Scales

Following a principal component analysis (PCA, oblimin rotation) of 112 participants with a complete and unimputed dataset of the HDQoL, stringent parallel analysis was conducted to identify three robust primary factors with eigenvalues greater than factors derived from the randomly generated parallel dummy datasets. A forced three factor solution (Table 2) showed minimal cross-loading and identified the following primary factors, i.e. (i) Primary Physical and Cognitive (PPC), (ii) Primary Emotions and Self (PES), and (iii) Primary Services (PSR).

**Table 2:** Three Primary Scales: factor loadings for the HDQoL

	Factor loading (Primary Scales)		
	
Items	Physical and Cognitive	Emotions and Self	Services
7. Dressing	0.95	—	—
3. Walking	0.94	—	—
8. Swallowing	0.93	—	—
9. Eating	0.90	—	—
4. Jobs around the house	0.87	—	—
6. Hobby	0.87	—	—
21. Remember date	0.81	—	—
19. Organize day	0.81	—	—
10. Operate television	0.79	—	—
27. Independence	0.79	—	—
2. Balance	0.77	—	—
14. Slow	0.77	—	—
13. Multitask	0.75	—	—
1. Carrying things	0.72	—	—
16. Concentration	0.71	(0.36)	—
5. Weight	0.71	—	—
18. Everyday memory	0.69	(0.33)	—
20. Follow conversation	0.64	(0.34)	—
15. Use words	0.64	(0.34)	—
17. Decision making	0.62	(0.41)	—
31. Role in family	0.54	(0.31)	—
25. Motivation	(0.48)	0.48	—
11. Tired	(0.46)	0.42	—
12. Sleep	(0.39)	0.38	—
24. Hope	—	0.81	—
29. Low mood	—	0.76	—
23. HD worry	—	0.75	—
32. Financial concerns	—	0.71	—
22. HD family worry	—	0.68	—
33. Irritated	—	0.64	—
34. Temper	—	0.62	(0.34)
30. Personal wishes	(0.38)	0.53	—
26. Get on with life	(0.49)	0.53	—
28. Confidence	(0.48)	0.52	—
36. Other's attitude to HD	—	0.51	—
35. Socialize	—	0.46	—
40. Information on HD	—	—	0.87
38. Services for HD	—	—	0.84
39. Management of HD	—	—	0.83
37. Support	—	—	0.45

HD, Huntington's disease; HDQoL, Huntington's disease health-related quality of life questionnaire.

These three primary factors were then subjected to Rasch analysis (RUMM2020) to test the unidimensionality of these subscales in a manner less constrained by the limitations and assumptions of classical test theory. Initially, the three primary scales showed unsatisfactory residual mean values, and significant chi-squared item–trait interactions (p < 0.005) indicating poor model fit. Following rescoring and the reordering of thresholds, the PSR scale achieved model fit; three items (weight, everyday memory, and role in family) and two items (sleep and HD family worry) were removed from the PPC and the PES scales respectively. These adjusted and rescored primary scales also met the assumption of local independence, demonstrating three unidimensional scales, and no retained item showed differential item functioning for gender. All scales also showed good to moderate targeting of scale to sample (average mean person location value for PPC = −1.05, PES = −0.20, PSR = −1.20), scale reliability was high (person separation index for PPC = 0.97, PES = 0.91, PSR = 0.78, as was internal consistency, i.e. Cronbach's alpha for PPC = 0.96, PES = 0.89, PSR = 0.76).

#### Six Specific Scales

From the previous PCA (oblimin rotation) of the HDQoL, the commonly used Kaiser's criterion for factor retention of eigenvalues greater than 1 and Cattell's scree test were used to identify six specific factors, that were meaningful and clinically relevant (Table 3), i.e. (i) Specific Cognitive (SCG), (ii) Specific Hopes and Worries (SHW), (iii) Specific Services (SSR), (iv) Specific Physical and Functional (SPF), (v) Specific Mood State (SMS), and (vi) Specific Self and Vitality (SSV).

**Table 3:** Six Specific Scales: factor loadings for the HDQoL

	Factor loadings (Specific Scales)					
	
Items	Cognitive	Hopes and Worries	Services	Physical and Functional	Mood State	Self and Vitality
18. Everyday memory	0.88	—	—	—	—	—
19. Organize day	0.78	—	—	—	—	—
21. Remember date	0.73	—	—	—	—	—
16. Concentration	0.72	—	—	—	—	—
13. Multitask	0.71	—	—	—	—	—
14. Slow	0.70	—	—	—	—	—
20. Follow conversation	0.69	—	—	—	—	—
15. Use words	0.65	—	—	—	—	—
17. Decision making	0.62	—	—	—	—	—
6. Hobby	(0.56)	—	—	0.39	—	—
30. Personal wishes	0.45	—	—	—	(−0.37)	—
28. Confidence	(0.44)	—	—	—	−0.32	—
12. Sleep	(0.32)	(0.32)	—	—	—	0.31
22. HD family worry	—	0.85	—	—	—	—
23. HD worry	—	0.76	—	—	—	—
24. Hope	—	0.69	—	—	—	—
32. Financial concerns	—	0.58	—	—	—	—
36. Other's attitude to HD	—	0.53	—	—	—	—
38. Services for HD	—	—	0.88	—	—	—
39. Management of HD	—	—	0.87	—	—	—
40. Information on HD	—	—	0.87	—	—	—
37. Support	—	—	(0.51)	—	—	0.48
5. Weight	—	—	—	0.89	—	—
9. Eating	—	—	—	0.82	—	—
7. Dressing	—	—	—	0.78	—	—
3. Walking	—	—	—	0.72	—	—
10. Operate television	—	—	—	0.71	—	—
8. Swallowing	—	—	—	0.71	—	—
1. Carrying things	—	—	—	0.61	—	—
2. Balance	(0.34)	—	—	0.51	—	—
27. Independence	(0.32)	—	—	0.48	—	—
4. Jobs around the house	(0.46)	—	—	0.48	—	—
33. Irritated	—	—	—	—	−0.83	—
34. Temper	—	—	—	—	−0.79	—
29. Low mood	—	—	—	—	−0.49	(0.38)
35. Socialize	—	—	—	—	−0.43	—
26. Get on with life	(0.34)	—	—	—	—	0.52
31. Role in family	—	—	—	—	—	0.43
25. Motivation	(0.34)	—	—	—	−0.34	0.40
11. Tired	(0.31)	—	—	—	—	0.39

HD, Huntington's disease; HDQoL, Huntington's disease health-related quality of life questionnaire.

Following Rasch analysis, all scales were rescored to correct for disordered thresholds and to improve model fit. Items still showing deviation from the model were eliminated from the SCG scale (i.e. personal wishes) and SPF scale (i.e. weight). Retained items did not show differential item functioning for gender. These six adjusted and rescored specific scales met the assumption of local independence and were unidimensional. All scales also showed good to moderate targeting of scale to sample (average mean person location value for SCG = −0.42, SHW = −0.25, SSR = −0.81, SPF = −1.23, SMS = −0.79, SSV = −0.65); scale reliability was high (person separation index for SCG = 0.97, SHW = 0.77, SSR = 0.82, SPF = 0.94, SMS = 0.86, SSV = 0.88, as was internal consistency, i.e. Cronbach's alpha for SCG = 0.94, SHW = 0.72, SSR = 0.77, SPF = 0.91, SMS = 0.78, SSV = 0.78).

#### Summary scale

Rasch analysis was used to identify global health-related quality of life from the 40 patient-derived items. The initial model fit was examined and the sources of model misfit addressed by rescoring and ordering thresholds, eliminating items (i.e. 1, 2, 4–6, 8, 12–17, 23, 26, 27, 30, 34, 36, 40 from Table 2) showing deviation from the model expectations or differential item functioning. For the remaining 21 items, the summary scale showed good targeting of scale to sample (average mean person location value = −0.69); scale reliability was high (person separation index = 0.92, as was internal consistency, i.e. Cronbach's alpha = 0.89).

### Psychometric properties of the HDQoL scales

The acceptability and psychometric properties of the three sets of scales (Primary, Specific and Summary) derived from the HDQoL are presented in Table 4. Item scores in each scale were summed and transformed into a scale ranging from 0 to 100, where higher scores indicate better health-related quality of life.

**Table 4:** Psychometric properties of the three sets of scales derived from the HDQoL

Sets of scales derived from the HDQoL	Mean score	Standard deviation	Floor effect (0–10%)	Ceiling effect (90–100%)	Skewness	Cronbach's alpha	Test–retest reliability
*Three Primary Scales*							
Physical and Cognitive	63.4	32.3	7.9	30.9	−0.4	0.9	0.8
Emotions and Self	53.2	27.2	7.2	10.5	−0.1	0.9	0.8
Services	78.7	28.9	3.9	50.0	−1.3	0.8	0.7
*Six Specific Scales*							
Cognitive	55.7	33.2	12.5	27.0	−0.1	0.9	0.8
Hopes and Worries	52.9	27.5	5.9	11.8	−0.1	0.7	0.7
Services	73.4	32.9	7.9	50.0	−1.0	0.8	0.7
Physical and Functional	68.4	32.0	7.2	36.2	−0.6	0.7	0.8
Mood State	61.7	27.8	7.2	20.4	−0.5	0.8	0.7
Self and Vitality	60.0	26.3	5.3	12.5	−0.5	0.8	0.7
*One Summary Scale*							
Summary Scale	61.0	24.8	4.6	16.4	−0.8	0.9	0.7

HDQoL, Huntington's disease health-related quality of life questionnaire.

All three sets of scales showed good stability over repeated testing (test–retest reliability ≥0.7), and good scale unidimensionality (Cronbach's alpha ≥0.7). Scale acceptability was generally good with minimal skewness (approximately between −1 and 1) and floor/ceiling effect (≤20%) for most scales. Since pre-symptomatic and at-risk participants were included in the sample, ceiling effects were therefore more apparent in the Primary Physical and Cognitive scale, and for both the Specific Physical and Functional as well as Specific Cognitive scales; however, there was no ceiling effect in the same sample for Primary and Specific scales that related to emotions and mood. The Services scale of both the Primary and Specific scales showed ceiling effect; which may naturally reflect the biased sample population who were recruited to this study via the HD association, and may therefore have been already particularly aware and satisfied with getting access to information, services and sources of support.

Construct validity was established by examining the three sets of scales with respect to two well-known generic instruments, the SF-12v2 and EQ-5D. Table 5 shows the expected pattern of correlations with each set of HDQoL-derived scales.

**Table 5:** Convergent and discriminant validity of the three sets of scales derived from the HDQoL, the three primary scales, the six specific scales and the single summary scale

	Three Primary Scales			Six Specific Scales						Summary
			
	Physical and Cognitive^b^	Emotions and Self^b^	Services^b^	Cognitive^b^	Hopes and Worries^b^	Services^b^	Physical and Functional^b^	Mood State^b^	Self and Vitality^b^	Summary Scale^b^
*EQ-5D*										
Mobility^a^	−0.7	−0.5	−0.3	−0.6	−0.3	−0.3	−0.7	−0.4	−0.5	−0.6
Selfcare^a^	−0.8	−0.5	−0.3	−0.6	−0.4	−0.3	−0.8	−0.4	−0.5	−0.7
Activity^a^	−0.8	−0.5	−0.4	−0.8	−0.3	−0.4	−0.8	−0.5	−0.6	−0.7
Pain^a^	−0.4	−0.4	−0.4	−0.4	−0.2	−0.4	−0.4	−0.4	−0.4	−0.4
Anxiety^a^	−0.4	−0.6	−0.2	−0.5	−0.5	−0.2	−0.3	−0.6	−0.5	−0.5
Index score^b^	0.8	0.7	0.5	0.7	0.5	0.5	0.8	0.6	0.7	0.8
VisAnalog^b^	0.7	0.5	0.4	0.6	0.3	0.3	0.6	0.4	0.6	0.6
*SF12v12*										
Physical^b^	0.8	0.4	0.3	0.7	0.2	0.3	0.8	0.3	0.5	0.6
Mental^b^	0.5	0.8	0.4	0.6	0.6	0.3	0.4	0.7	0.7	0.7

HDQoL, Huntington's disease health-related quality of life questionnaire.

a Higher score indicates lower quality of life/health status.

b Higher score indicates better quality of life/health status.

## Discussion

Using a data-driven approach, this article presents a novel disease-specific patient-reported health-related quality of life outcome measure for HD, which is called the Huntington's Disease health-related Quality of Life questionnaire (i.e., HDQoL). Both traditional and Rasch analyses were used to derive three sets of scales derived from this instrument, i.e. (i) three robust Primary Scales that represent core factors underlying health-related quality of life in HD, (ii) six Specific Scales that represent a clinically meaningful HD-specific profile of pertinent aspects of health-related quality of life, and (iii) a Summary Scale. The administration of the HDQoL in the target user group, using a substantial and diverse sample of HD patients, allowed its performance to be evaluated to determine its suitability as a measure of health-related quality of life. The HDQoL showed good test–retest reliability, construct validity, and scale acceptability. Its scales are appropriately unidimensional, and have acceptable measurement and psychometric properties given the broad spectrum of patients and participants in this sample. The three Primary Scales and single Summary Scale are robust and promising outcome measures for evaluating health-related quality of life in HD patients, and the six Specific Scales offer the potential for examining more detailed and clinically meaningful aspects of quality of life in this population. Importantly, the patient-derived HDQoL was found to be acceptable to people living with HD in terms of its content and administration time; two thirds of this diverse spectrum of patients, including late-stage HD, completed this 40-item questionnaire in less than the mean completion time of 22 min.

Our sample comprised more women than men, and although this is unlikely to have significantly influenced the results given what is known of HD and also gender comparison in quality of life in Parkinson's disease (12), further work to replicate these results in a larger sample is warranted. This will also allow limitations of this study to be addressed, such as the possible sample self-selection bias, and investigation of other clinical variables including clinician rating of patient severity rather than inference from self-report. While a patient-reported measure of health-related quality of life is necessarily subjective, it would also be informative to examine proxy (e.g. carer) ratings of patients' health-related quality of life in view of the cognitive impairment that is associated with HD. Further work is also needed to examine issues such as scale responsiveness and sensitivity to placebo effects. These next steps will provide further information regarding the instrument and its subscales, and will be useful for future clinical applications.

In summary, the patient-derived HDQoL is a new disease-specific questionnaire which provides appropriate measures of health-related quality of life to capture the true impact of HD across disease severity. The HDQoL provides validated scales which are HD specific and therefore more likely to be sensitive than generic scales. It allows the impact of interventions to be appropriately and holistically evaluated from the patients' perspective, and will be valuable in promoting more fully informed decision making in the management of HD.
